# Supplementary material for: Chromothripsis during telomere crisis is independent of NHEJ, and consistent with a replicative origin
Source: Genome Res. 2019 May;29(5):737–49. doi: 10.1101/gr.240705.118 (PMC6499312; doi:10.1101/gr.240705.118)
Supplement: Supplemental Material [file supp_gr.240705.118_Supplemental_file_1.zip › contigs/annotated_contigs/DB76/contig.2.DB76_length_313_mean_cov_8.70607028754.docx]

**DB76_length_313_mean_cov_8.70607028754**

```
AAACACAAAAAAAA|AAGACACACACACAGAGAGAGAGAGAGAGACAGAGACAGAAAGAAAGACAAAGACAGAGAGAGAGAGAGAGAGA
               >chrUn_gl000220:136186-136265 - E=4e-33 p=2e-02
GAG|AG|CAAAAGTAGACGTGTTTTAGGTCTTCATGAGCTTAGTCAAGTGGAGCATGCCAATGGCAAACAAATCAATATGTGATTACAG
    >chrX:96866346-96866572 + E=2e-113
TTGTGTTTAGTGCCTTAGAAGGAAAGACTGCAGATATTGTAGGAGAAAATAATGGTGTGAGAGAGAGTGTGTGTGTGAGTGTGTGTGTG

AGTGTGTGTGTGTGAGTGTGTGTGTGTGTGTGTGTGTGTGGTGGAAAGG
```
